# InDEL instability in two different tumoral tissues and its forensic significance

**DOI:** 10.1007/s12024-024-00808-5

**Published:** 2024-04-03

**Authors:** İpek Gürel, Faruk Aşıcıoğlu, Gökhan Ersoy, Özlem Bülbül, Tülin Öztürk, Gönül Filoğlu

**Affiliations:** 1 Department of Science, Institute of Forensic Sciences and Legal Medicine, İstanbul University- Cerrahpaşa, İstanbul, Türkiye; 2 Department of Molecular Biology and Genetics, Faculty of Arts and Sciences, Haliç University, İstanbul, Türkiye; 3 Department of Medical Sciences, Institute of Forensic Sciences and Legal Medicine, İstanbul University-Cerrahpaşa, İstanbul, Türkiye; 4 Department of Medical Pathology, Cerrahpaşa Faculty of Medicine, İstanbul University- Cerrahpaşa, İstanbul, Türkiye

**Keywords:** InDels, Instability, Tumoral tissue, Identification, STR, Forensic genetics

## Abstract

There may be cases where malignant tumor samples can be used for forensic DNA profiling studies. STRs are the first systems preferred in forensic science laboratories for identification purposes. However, genetic instability in tumoral tissues causes STR polymorphism to change, leading to erroneous results. On the other hand, insertion/deletion polymorphism (InDels) are used as genetic markers in forensic science, as they have features that make both STR and SNPs preferable. Although previous studies approved that STR instability is observed in many different tumors, there are only a few studies that have displayed the instability of InDels in tumoral tissues before. In this study, it was aimed to determine whether instability is observed in formalin-fixed paraffin-embedded breast and thyroid tumoral tissues at 36plex InDel Panel. A total of 47 cases, 26 of which were diagnosed as breast cancer and 21 as thyroid cancer, were included in the study. In 21 of 26 (80.76%) breast cancers mutational changes were observed, however only 6 of 21 (28.57%) thyroid carcinoma cases displayed instability.Moreover, in these six cases, mutations were detected at only 1 or 2 loci. The most common change in both tissues was loss of heterozygosity. These findings suggest that paraffin embedded tissues of thyroid tumor can be used in cases of forensic genetic identification, however paraffin embedded breast cancer tissues should be examined with care. In conclusion, low InDel mutation rates compared to STR instability, make InDel analysis from paraffin blocks suitable for forensic genetic identification. However, researchers should keep in mind that there may be differences between the profiles of the tumoral tissues taken as reference and the actual case. In addition, by incorporating additional markers such as SNPs and microhaplotypes with low mutation rates into the study alongside Indels, researchers can significantly enhance the discrimination power in identification processes.

**Supplementary Information:**

The online version contains supplementary material available at 10.1007/s12024-024-00808-5.

## Introduction

Analysis of DNA polymorphisms in humans is a valuable tool for forensic identification, if there is a reference sample to compare with the sample in question. Where there is no reliable reference sample, it is necessary to find an alternative source such as an histopathology specimen. Some difficulties may arise when the accuracy of the reference sample is questionable with possible genetic variability, such as malignant tumors [1]. This is because tumor tissues generally have mutations, which may affect genotype analysis. However, there are some situations where the use of tumor tissues may be the most advantageous option [2–4].

The reason why STR loci are the first preferred method in forensic science laboratories is that there are approximately 1 million STR loci in the genome, and they show high polymorphism due to their different repeat numbers and content of these repeats and thus have high discrimination power [5–9]. STRs are spread in non-coding regions of the genome and contain a higher mutation rate than other genomic regions. This is because DNA polymerase shifts during DNA replication and repair, causing the deletion or insertion of repeats in STRs. This change is defined as microsatellite instability (MSI) [10, 11]. Studies have shown that genetic instability is a very common phenomenon observed in many different tumors [12–16]. It is known that tumor DNA harbors genetic changes not only in defined genes but also in repetitive DNA sequences. Loss of heterozygosity (LOH) and MSI are seen as genetic instability in many tumors [3]. In addition to the mutative effects of tumors, formalin-fixed paraffin-embedded (FFPE) processes of archived tumoral tissues used in the analysis itself may cause degradation of the genetic material. The most important reason for this effect is that the tissues undergo chemical preparation processes that will lead to degradation before they are embedded in paraffin [17, 18].

InDels are one of the most common polymorphisms resulting from the insertion or deletion of one or more nucleotides in the human genome [19]. InDels contain much lower mutation rates compared to STRs, and also have a shorter amplicon length (60–200 bp). This makes it possible to use InDels in degraded samples [20]. In addition, it has been stated that InDels can be a potential genetic marker in forensic sciences due to their abundance in the genome and ease of analysis [21].

The aim of this study is to observe whether the instability is seen in tumoral tissues at 36plex InDel Panel [22]. For this purpose, InDel genetic profiles obtained from tumoral and non-tumoral tissues were compared. The determination of whether specific loci exhibit mutations or remain in their normal state can be made by contrasting the identified genetic changes in tumoral tissues with their corresponding normal counterparts. According to our knowledge, this is the first study to question the existence of genomic instability in InDels for forensic purposes using FFPE tissues.

## Materials and methods

### Sample collection

A total of 47 cases, 26 of which were diagnosed as breast cancer and 21 as thyroid cancer, were included in the study. A total of 94 samples containing both tumoral and non-tumoral tissues, two paraffin blocks for each case, were selected and studied. As we were unable to reach these individuals during their active illness, we couldn’t take fresh biological tissues as a control group. The samples were collected from the archive of Istanbul University-Cerrahpaşa, Cerrahpaşa Faculty of Medicine, Department of Medical Pathology. Informed consent was obtained by contacting the owners of the samples. The ethical permission was approved by Clinical Research Ethics Evaluation Committee at İstanbul University-Cerrahpaşa, Cerrahpaşa Faculty of Medicine on March 2nd ,2021 (No. 17,853).

The range values for the storage time of FFPE tissues were determined as 2–29 months (Median = 8 months) in cases with breast carcinoma and 1–38 months (Median = 19 months) in cases with thyroid carcinoma. In one case with breast tumor, the age, grade and storage period of the blocks could not be determined because the final report was not available. No significant difference was found between the breast and thyroid tumor groups in terms of block storage times. (Mann Whitney U = 334, *p* = 0.114).

The age range of the cases was between 28 and 77 for breast cancer cases (mean = 53.1 ± 10.7), 28–63 (mean = 46.9 ± 9.8) for thyroid cases. Accordingly, the average age of the thyroid carcinoma patient group is statistically significantly lower than that of breast cancer cases (Mann Whitney U = 171.500; *p* = 0.045). Pathological diagnosis of all thyroid tissues was papillary thyroid carcinoma. Although different types of cancer were seen in breast tissues, most of them were invasive ductal carcinomas. Types of breast carcinomas are given in Table 1. The grades (breast) and thyroid types (thyroid) of the cases were recorded by reviewing their reports. Accordingly, 11 (44%) out of 26 breast cancer cases were classified as grade 2, and 14 (56%) were classified as grade 3. In one case, age, grade, and block storage period could not be determined because a report could not be obtained. For thyroid cancer cases, 1 (5.5%) was determined as Stage 1, 7 (38.9%) as Stage 2, and 10 (55.6%) as Stage 3. The stage could not be determined in the reports of 3 cases. Age and grade/Stage information for the cases are provided in Supplementary Table 1.

**Table 1:** Pathological diagnoses of cancer tissues

**Pathological Diagnosis**	**n**
Thyroid Papillary Carcinoma	21
Invasive Ductal Carcinoma	17
Mixed Carcinoma I (Invasive Ductal + Invasive Lobular Carcinomas)	4
Mixed Carcinoma II (Invasive Ductal + Invasive Colloidal Carcinomas)	3
Invasive Lobular Carcinoma	1
Metaplastic Carsinoma	1

### Sample preparation and DNA extraction

Sections of 2 μm thickness were prepared from paraffin blocks using a microtome. Slides were stained with hematoxylin-eosin dye using the standard method [23]. Hematoxylin-eosin-stained slides were examined by the pathologist under a light microscope to distinguish paraffin blocks containing tumor and non-tumor tissue.

DNA was extracted from both tumor and non-tumor tissue sections by using E.Z.N.A. Tissue DNA Kit (Omega Bio-Tek, Norcross, GA, USA). Before DNA isolation, tissues were deparaffinized using xylene and sequential alcohol solutions of decreasing concentration according to the kit protocol. DNA samples were quantified using the Quant-iT dsDNA High-Sensitivity (HS) Assay Kit (Invitrogen, CA, USA) by Qubit fluorometer (Invitrogen, CA, USA).

### PCR amplification

In this study, 36-InDelplex panel developed by Filoğlu et al. was used for genotyping, and PCR was carried out according to the procedure of this study [22]. In tumoral and the non-tumoral tissues, PCR was performed using 34 InDel loci located on autosomal chromosomes (rs34660708, rs34495360, rs2308135, rs2307789, rs2307521, rs2308112, rs2308163, rs1610919, rs2308137, rs16646, rs144389514, rs56168866, rs16671, rs33972805, rs25549, rs16722, rs2067304, rs140861207, rs1160981, rs4646006, rs6480, rs28369942, rs2307838, rs3062629, rs10590424, rs16458, rs10623496, rs1610937, rs16363, rs2067147, rs1160965, rs2307656, rs2308101, rs2308072, rs2067191), as well as the InDel locus on the Y chromosome (rs2032678) and the amelogenin locus (AMG-XY).These InDel loci are part of the 36-InDelplex panel developed by Filoğlu et al., demonstrating high polymorphism in the Turkish population and their applicability in forensic identification [22]. PCR was performed using the SimpliAmp™ Thermal Cycler (Thermo Fisher Scientific, MA, USA) under the following conditions: initial denaturation at 95 °C for 11 min, 30 cycles of denaturation at 94 °C for 20 s and amplification at 62 °C for 3 min, followed by elongation at 60 °C for 1 hour. The components used in PCR are 4.2 µl Master Mix, 0.5 µl Taq Polymerase, 3 µl Primer Mix and 3 µl DNA, with a total volume of 10.7 µl.

Complete profiles were obtained with a single PCR in most of the samples. However, PCR was repeated for a small number of samples to eliminate missing alleles, especially in some non-tumoral breast tissues. Specifically in some samples, DNA was initially diluted to a concentration of 1 ng/µL and added to the PCR mixture such as in Filoğlu et al’s study [22], and the full profile was obtained. However, in some other cases, profiles containing many missing alleles were obtained despite using up to 3ng/µL DNA input. In these cases, the PCR protocol was repeated by adding the DNA undiluted to the PCR mixture to obtain a complete profile.

### InDels genotyping

Electrophoresis of PCR products was performed by using an eight capillary Applied Biosystems™ 3500 Genetic Analyzer (Thermo Fisher Scientific, MA, USA). First, a mixture containing 9.5 µl of Hi-Di™ Formamide and 0.5 µl of GeneScan™ LIZ 500 Size Standard for each sample was prepared and vortexed. Afterward, 10 µl of the mixture and 1 µl of PCR product per sample were loaded into the plate well. Samples were in the G-5 module of GS STR POP7 (1 ml) with 36 cm capillaries, using POP-7 polymer; the injection was carried out at 1.2 kV and 24 s, 60 °C for 30 minutes.

GeneScan™ LIZ 500 Size Standard was used to verify the running conditions and detect InDel loci. After verifying the position of this standard for each sample, the locations of InDels were determined by comparing them with the standard reference(K562). The genotypes in the electropherogram were evaluated using GeneMapper Software v.5.0 (Thermo Fisher Scientific, CA, USA).

InDel instability is defined as the emergence of a new allele or the replacement of an existing allele. For InDel markers, InDel instability is observed when the homozygous genotype observed in healthy tissue transforms into either heterozygosity or a distinct homozygous state in the tumor tissue. Furthermore, allelic deletion detected in the tumor tissue, into its corresponding non-tumoral tissue, was categorized as loss of heterozygosity.

## Results

Since the analysis kit consists of 36 loci, 936 loci in 26 breast cancer cases and 756 loci in 21 thyroid cancer cases were analyzed. If no allele peaks were observed at a locus, or if there was only one allele with a height not exceeding analysis thresholds (200 Relative Fluorescence Unit), loci were not included in the analysis. Profile could be obtained in all 47 cases. The number of loci that could not be analyzed after this evaluation is given in Table 2. As a result, 815 loci of breast and 685 loci of thyroid cancer cases could be compared. There was no statistically significant difference between the tissue group variables (tumor vs. non-tumoral block; χ2 = 4,2128, *p* > 0,05) and the cancer type variables (breast vs. thyroid; χ2 = 2,209, *p* > 0,05) in terms of degradation rates.

**Table 2:** The table shows the degradation rates at all analyzed loci and the number of loci that were successfully compared

	**Breast cancer cases**			**Thyroid cancer cases**		
The number of…	Non-tumoral tissues	Tumoral tissues	Total	Non-tumoral tissues	Tumoral tissues	Total
loci included in the analysis	936	936	1872	756	756	1512
excluded loci due to degradation n[% of total loci]	63	79	142 (7,58%)	50	44	94 (6,21%)
loci that show degradation in both tumoral and control tissue in the same person	21			23		
loci that can be evaluated	873	857	1730	706	712	1418
pair of loci successfully compared	815			685		

For all 47 cases, the median value of cases with undetectable alleles, due to degradation, was determined as 1.5 when examining the distribution of 192 loci for each locus (Range 0–28). Accordingly, degradation-related undetectable cases are observed in only 15 out of 36 loci, with 3 or more cases, and 8 of these loci exhibit degradation in 10 or more cases. The numerical distribution of loci where 3 or more cases have undetectable alleles due to degradation is provided in Table 5. While the most significant allele loss is observed in ID13(rs144389514), there has been no allele loss due to degradation in loci ID26(rs2308163), ID3(rs2307521), ID16(rs16458), ID32(rs2308101), ID9(rs1610937), ID17(rs3062629), ID31(rs16671), ID27(rs2067147), ID15(rs16646), ID24(rs1610919), ID30(rs34495360), ID25(rs34660708), and ID29(rs25549).

### Mutation rates of loci for both tumor types

The changes were observed in 75 of 815 loci in breast tissues (9,2%), of which %65.3 complete loss of heterozygosity, %20 of InDels instability, and %14,7 partial loss of heterozygosity. Whereas changes in thyroid cases were observed in 10 out of a total of 685 loci (%1,5). Distribution of changes were 3 (30%) complete, 3 (30%) partial loss of heterozygosity, and 4 (40%) InDels instability among these changes. In the point of tumor types, mutations causing genetic instability are shown in Table 3.

**Table 3:** Number of mutations found in each InDels locus by tumor type (pLOH: Partial loss of heterozygosity; cLOH: Complete loss of heterozygosity MSI: Microsatellite instability)

	**Breast Tissue Samples**			**Thyroid Tissue Samples**			**Total**
**Loci**	pLOH	cLOH	MSI	pLOH	cLOH	MSI	
**ID26**	2	3	2				7
**ID3**		2	1				3
**ID16**	3	4				1	8
**ID32**	1	3	1				5
**ID9**	1	2	1				4
**ID37**		2					2
**ID10**		2	2				4
**ID27**	1	2					3
**ID15**		1					1
**ID24**	1	6	1	1			9
**ID5**	1		2			1	4
**ID34**		2		2	1		5
**ID11**		1					1
**ID33**		1					1
**ID23**		2			1		3
**ID14**					1	1	2
**ID22**		1	1				2
**ID30**		3					3
**ID1**		3				1	4
**ID17**			2				2
**ID28**		1					1
**ID25**	1	1					2
**ID21**		2	1				3
**ID29**		1					1
**ID12**			1				1
**ID18**		3					3
**ID20**		1					1
**Total**	11	49	15	3	3	4	75 + 10 = 85

**Fig. 1:**
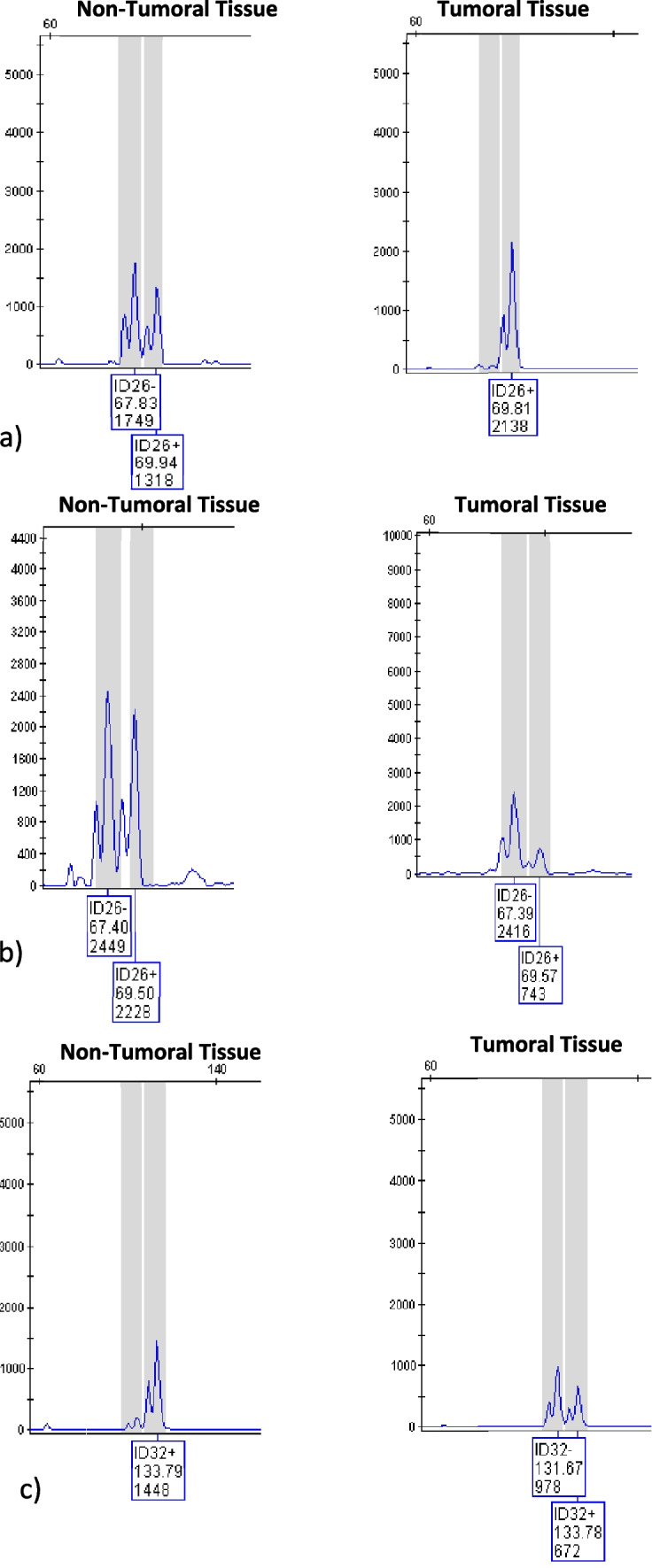
The type of instabilities on InDel alelles of non-tumoral and tumoral tissues. **a** Complete loss of heterozygosity at ID 26 locus, **b** Partial loss of heterozygosity at ID 26 locus, **c** Microstallite instability at ID 32 locus

The most changed loci are in order of frequency ID24, ID16 and ID26 loci. Examples of complete loss of heterozygosity, partial loss of heterozygosity and InDels instability were given in Fig. 1a, b and c, respectively.

According to given rates at Table 3, the incidence of InDels mutations in breast cancer tissues is significantly higher than in thyroid cancer tissues (Chi-square = 40,4694; *p* < 0.001). On the other hand, there was no statistically significant difference between the two cancer/tissue types in terms of distribution according to mutation types (Chi-square = 4.647; *p* > 0.05).

According to the Spearman correlation test results, no correlation was identified between the number of mutations detected in the cases and their ages (*r* = 0.038, *p* = 0.800) or block storage times (*r* = -0.045, *p* = 0.764). Furthermore, it was determined that the number of mutations did not exhibit a statistically significant difference between different grades in breast cancer and different stages in thyroid cancer (Mann Whitney U = 74, *p* = 0.893; Kruskal-Wallis = 571, *p* = 0.752, respectively).

### Efficiency of comparison on a case-by-case basis

Mutational changes were observed 21 of 26 (80.8%) breast cancer cases whereas only 6 of 21 thyroid carcinoma expressed these changes (28.6%). Number of breast cancer cases with mutational changes was significantly higher than thyroid cancers (Fisher exact test; *p* < 0.001). Although the number of mutant loci was ranged from 1 to 10, 12 of 21 breast cancer cases had two or less than two mutations. On the other hand, the number of mutant loci was 3 in one of the six mutated thyroid cancer cases whereas 1 or 2 mutations were observed in the other five cases. Distribution of overall and detailed numbers of mutations on a case-by-case basis was given at Table 4 and Supplementary Table 1.

**Table 4:** Number of mutant loci on a case-by-case basis

**Number of mutant loci**	**Tumoral Tissue(n)**		**Total**
	**Breast**	**Thyroid**	
0 locus	5	15	20
1 locus	8	3	11
2 loci	4	2	6
3 loci	1	1	2
5 loci	1	0	1
6 loci	2	0	2
7 loci	3	0	3
8 loci	1	0	1
10 loci	1	0	1
Total	26	21	47

## Discussion

InDels can be used as a genetic marker in forensic sciences [12, 16]. They are very common in the genome, provide successful results in degraded samples due to their small amplicon size, and have a lower mutation rate than STRs. The methods used for the analysis of STRs can also be used for InDels analysis. Therefore, InDels have been observed to be more expedient than STRs [13, 16, 24].

Loss of heterozygosity is characterized by mutation of one allele followed by deletion of the remaining allele [15]. In addition, the loss of heterozygosity also differs according to the amount of peak intensity. To be considered as complete loss of heterozygosity (cLOH), the peak intensity should be less than 0.5 and higher than 2, while partial loss of heterozygosity (pLOH) occurs when the peak size of an allele decreases by more than %50 [2, 25].

Effect of the paraffinization process on DNA analysis has been investigated in various studies before. In one recent example, Vitosevic showed that 3 gene regions, which are often used as reference genes in genetic analysis studies, can be amplified in paraffin blocks for up to 30 years [26]. Indeed, the fixation and embedding process causes degradation of DNA due to its fragmentation and chemical modifications [27, 28]. These modifications may occur due to mechanisms such as tissue aging over time in the fixative, hydrolysis of DNA with formic acid, and formation of intramolecular methylene bridges [29]. Covalent cross-links formed during fixation can interfere with extraction and downstream applications, impacting PCR, sequencing, and molecular analyses. Additionally, the fixation process often reduces overall DNA yield, posing challenges in high-throughput applications or when working with limited tissue samples. These limitations highlight the complexity and constraints associated with using FFPE tissues for DNA studies [30, 31]. In our study this degradation effect manifested itself as a randomly distributed reduction in peak sizes or complete loss of alleles in some of the InDel loci both in tumoral and non-tumoral blocks, as well.

In many studies conducted with paraffin embedded blocks, it is reported that there is a decrease in the amount of DNA as well as in the quality of DNA [27, 28]. In order to cope with the disadvantages of using tissues embedded in paraffin blocks, procedures such as adjusting the coverage numbers, the amount of DNA template analyzed, pretreatment methodologies, optimizing proteinase K digestion conditions or simplifying DNA extraction procedures can be implemented [32, 33]. Kerick et al. showed that there was no difference between FFPE and snap frozen tissues in terms of detection of InDel mutations because of increasing the coverage levels to 40 × [34]. In one study, they reported that successful results were obtained by adding 30 ng of DNA template to the PCR reaction [35]. In our study, this problem is solved by increasing the amount of DNA. In certain samples, diluting DNA to 1 ng/µL, following Filoğlu’s study [22], yielded a complete profile, while in other cases, profiles with numerous missing alleles persisted even with 2 ng/µL or 3 ng/µL DNA. In these cases, the PCR and electrophoresis protocol was repeated by adding the DNA undiluted to the PCR mixture to obtain a complete profile. This adjustment proved effective because, even during fixation, certain DNA chains endure in an intact state. The increase of template DNA quantity enhances the likelihood of amplifying intact DNA chains, thereby achieving a more comprehensive and accurate profiling of the genetic material. In these cases, a complete profile could be obtained by adding DNA without dilution. As a result, an overall profile of 88.7% could be obtained in tumor and non-tumor tissues.

From another perspective, although they show a certain degree of degradation, FFPE tissues are attractive tools for clinical studies because they are much easier to obtain than frozen tissues. However, this degradation effect may not cause a limitation as expected [19]. Although Oh et al. obtained lower yielding and mapping results in FFPE samples compared to frozen samples in their exome sequencing studies, they showed that this did not have a statistically significant effect. Moreover, they showed that frozen samples could lead to higher off-target rate determinations compared to FFPEs, thus pointing to an advantageous aspect for FFPE tissues. They attribute this to DNA fragments that shorten with fragmentation and say that this will increase on-target coverage [36]. This study’s primary aim is to determine whether the identification success of the InDel multiplex panel exhibits any difference between tumor tissues and normal tissues. In this retrospectively conducted study, the use of paraffin blocks as controls could have quickly demonstrated differences between normal and tumoral tissues. Indeed, in a study similar to ours, Oliveira and colleagues utilized tumor-free tissue within the tumor tissue block as a control [35].

There was no significant difference between the degradation rates in terms of two tumor types we studied or whether the tissue contains tumoral or non-tumoral tissue. These ratios, which were between 6.2% and 7.5% in both cases (See Table 2). Similarly in a study by Soo et al., small-sized DNA or RNA fragments were amplified successfully in formalin-fixed paraffin-embedded samples after they were kept for several years [37]. Conversely, in a study by Guyard et al., they show that after 4 to 6 years the strong decrease in the amount of amplifiable DNA is due to fragmentation of DNA [38]. In our study, there were medium-long waiting times distributed between 1 and 38 months compared to these studies, and more than 90% matching was possible (Supplementary Table 1).

As reported at "Results" section, the mutations were detected in only six of 21 thyroid cancer cases. Moreover, in these six cases, mutations were detected at only 1 or 2 loci. This finding suggests that archival FFPE tissues of thyroid tumor can be used in cases of forensic genetic identification. However, the presence of mutations in 21 of the 26 breast cancer cases examined suggests that paraffin blocks, in which this cancer type is detected, should be used carefully in forensic genetic identification. The most mutations observed in the ID24(rs1610919), ID26(rs2308163), and ID16 loci(rs16458), along with the prevalence of cLOH, results from these loci should be treated with suspicion. Particularly in malpractice cases, it is essential to acknowledge that such variations in these loci can be dismissed. However, as reported by Nam SK et al., depending on the nature of the case, it is still possible to make a reliable comparison in cases with a small number of mutations [37]. In a study where Pereira et al. used 38 non-coding bi-allelic indel markers on different populations, they found random match probabilities (RMP) to be 1 in 10-17 billion even for the 25 most informative markers. Accordingly, even if an incomplete profile is obtained, the possibility of identification is high [19].

Turajlic et al. reported that mutations at renal cell carcinomas are twice as much as all other cancer types and breast cancers have higher averages than thyroid cancers in terms of both InDel numbers and InDel ratio [39]. Similarly, we observed higher instability rates in breast cancer cases than in thyroid cancer cases. On the other hand, we found that the number of mutations did not show a relationship with the grade for breast cancers or the stage for thyroid cancers. Moreover, no difference was detected according to the storage time of the blocks and the age of the cases. In this instance, it does not seem possible to say with certainty, type of malignancy may play an important role in the rate of mutational changes. Indeed, Wu et al. suggested that the number of InDel mutations which is described as ‘Tumor Mutational Burden (TMB)’ may differ between cancers and associated with prognosis and treatment response [40]. Unlike this study, which took into account the coding regions, our study showed the effect of cancers on the analysis in terms of forensic identification, as it was performed on non-coding loci.

An additional significant result indicated by our findings is that, when identification is required using an InDel panel from FFPE tissues, it is preferable to extract material from a non-tumor block rather than a tumor block. No similar study has been reported in the literature. In the closest study to ours, Oliveira et al. detected InDel mutations in the CDH1 gene that were not present in normal tissue in cases of Hereditary Diffuse Type Gastric Carcinoma [35]. In their study, they dissected and compared both tumor and non-tumor tissue from the same FFPE block. Although their study focused on a gene that codes differently from ours, our findings underscore the preference for selecting these blocks whenever normal tissue is available. Tumorous FFPE tissues may be utilized in samples such as needle biopsy where no normal tissue block is present. It is essential to determine the mutation dynamics in cancer types for this to be possible.

Cancerous transformation in tissues may adversely affect the success of forensic identification studies. It has been shown by previous studies that cancer can accompany disruptions in STR loci [17, 41, 42]. For example, Peloso and his colleagues worked with archival FFPE tissues taken from 24 people with lung cancer as well as corresponding normal tissues obtained from lymph node sections of the same patients. Although allele drop-out was observed in at least one STR in 20 of 24 samples, allelic imbalance was observed in the STR loci of all samples. Additionally, a small portion of the samples showed loss of heterozygosity [13]. Also, Anaian et al. observed DNA profiles from 12 gastric,12 breast and 10 colorectal formalin-fixed paraffin-embedded tissue (FFPET) samples, revealing 55 cases of partial loss of heterozygosity (pLOH), 15 cases of complete loss of heterozygosity (cLOH), and 13 cases of microsatellite instability (MSI) [2]. On the other hand, other identification markers (e.g. SNPs, InDels) have been also tested in tumor samples [25, 43]. In the study of Tozzo et al., 61 (92.4%) of 66 frozen tumor samples (hepatic, gastric, breast, and colorectal cancer) were found to have at least one mutation in InDel loci [25]. Majority of these mutations were LOHs of which 41.7% were only partial (pLOH) and MSI events constituted only 20.6% of all the mutations. These authors argued that because of these ratios, the use of STR loci for identification purposes would be more appropriate than InDel loci. On the contrary, in a study by Zhao et al., only pLOH and cLOH mutational events of InDels loci were observed in fresh tumoral tissue samples (colorectal and gastric cancer). The total mutation rate of InDels was 0.25% in tumoral tissues [43]. Authors suggested that InDels might be more powerful than STR in source identification; unlike the results reported by Tozzo et al.

Our results support the view that MSI is the least common type of InDel mutations in tumoral tissues. While only 22,35% of the mutations are of this type, the vast majority are LOH mutations. Moreover, in 27 of 47 cases, a mutation was detected in at least one of the loci compared to non-tumoral tissues (See Table 3).

A problem we are dealing with was more missing alleles in the profiles of the non-tumoral breast tissue samples which can be due to the high density of adipose tissue that leads to losses in terms of DNA yield. Similar to our study, in a study conducted by McDonough et al., it is stated that there is less DNA in non-tumoral breast tissues than the corresponding tumoral part [28]. In the study conducted by Mee et al., it was observed that since non-tumoral breast tissue consists of almost completely fat, it has less DNA, RNA and protein amount compared to tumoral breast tissue [44].

A limitation of our study is that it was carried out retrospectively on archival FFPE tissues. The degradation effect of paraffinization will be able to be determined more clearly in future studies using fresh biological tissues as the control group. In prospectively planned studies, the effects of prognostic variables regarding the tumor on both degradation and detection of mutations can be determined. The effectiveness of the panel can be increased by determining tumor-specific mutation distributions (Table 5).

**Table 5:** Unmatched loci and case numbers

*n	**Loci**
**3 cases**	ID8(rs2067304), ID10(rs10590424)
**4 cases**	ID11(rs1160981), ID21(rs2308112)
**6 cases**	ID18(rs2308072)
**7 cases**	ID33(rs6480)
**9 cases**	ID17(rs3062629)
**10 cases**	ID1(rs4646006)
**11 cases**	ID20(rs56168866)
**13 cases**	ID35(rs2032678)
**15 cases**	ID7(rs2307656)
**17 cases**	ID4(rs28369942)
**21 cases**	ID28(rs16722)
**24 cases**	ID19(rs10623496)
**28 cases**	ID13(rs144389514)

As a result, complete profiles of 36 InDel loci were obtained in the majority of paraffinized tissues, both tumoral and non-tumoral. Multiple mutations were seen in some of the cases, while no mutations were found in almost half of the cases. In the vast majority of cases with mutations, only one or two mutations such as loss of heterozygosity, partial loss of heterozygosity and InDels instability were observed. These low InDel mutation rates compared to STR instability, make InDel analysis from paraffin blocks suitable for forensic genetic identification. However, researchers should keep in mind that there may be differences between the profiles of the tumoral tissues taken as reference and the actual case. In addition, by incorporating additional markers such as Single Nucleotide Polymorphisms (SNPs) and microhaplotypes with low mutation rates into the study alongside Indels, researchers can significantly enhance the discrimination power in identification processes. Low mutation rates reduce the likelihood of errors or inconsistencies in the analysis, ensuring the accuracy of the results over time. The combination of Indels, SNPs, and microhaplotypes creates a multi-marker approach that significantly boosts the discrimination power in identification processes. This increased power allows for more precise differentiation between individuals, particularly in forensic applications or population genetics studies.

## Key points

1. The 36-InDelplex panel was genotyped in 47 cases, 26 with breast cancer and 21 with thyroid cancer.
2. Mutational changes were observed in 75 (9.2%) of 815 loci in breast tumor tissue and 10 (1.5%) out of 685 loci in thyroid. While the incidence of InDels mutations in breast tumor tissues was significantly higher than in thyroid tumor tissues, there was no statistically significant difference between tumor/non-tumor tissue type.
3. Mutational changes were observed 21 of 26 (80.8%) breast cancer cases whereas at only 6 of 21 (28.6%) thyroid carcinoma cases.
4. Low InDel mutation rates compared to STR instability, make InDel analysis from paraffin blocks suitable for forensic genetic identification. However, researchers should keep in mind that there may be differences between the profiles of the tumoral tissues taken as reference and the actual case. In addition, by incorporating additional markers such as SNPs and microhaplotypes with low mutation rates into the study alongside Indels, researchers can significantly enhance the discrimination power in identification processes.
5. The reference samples should not only consist of tumor tissues, but also DNA should be obtained from the non-tumoral part of the paraffin block.

## Electronic supplementary material

Below is the link to the electronic supplementary material.

Supplementary Material 1 (DOCX 24.1 KB)
