# Supplementary material for: InDEL instability in two different tumoral tissues and its forensic significance
Source: Forensic Sci Med Pathol. 2024 Apr 3;20(4):1241–50. doi: 10.1007/s12024-024-00808-5 (PMC11790770; doi:10.1007/s12024-024-00808-5)
Supplement: Supplementary file 1 — Supplementary Material 1 (DOCX 24.1 KB) [file 12024_2024_808_MOESM1_ESM.docx]

**Supplementary table 1: Detailed distribution of number of mutations on a case-by-case basis.**

|  |  | **BREAST TUMORS** | | | | | | |  | **THYROID TUMORS** | | | | | | | |
| --- | --- | --- | --- | --- | --- | --- | --- | --- | --- | --- | --- | --- | --- | --- | --- | --- | --- |
|  |  | **Age** | **Grade** | **St** | **MSI** | **pLOH** | **cLOH** | **Total** |  | **Age** | **Stage** | **St** | **MSI** | **pLOH** | **cLOH** | **Total** |  |
| **BREAST TUMORS** | **Case 1** | 45 | 2 | 15 | 4 |  | 3 | 7 | **Case 27** | 42 | 3 | 2 | 0 |  | 0 | 0 |  |
|  | **Case 2** | 55 | 3 | 8 | 0 |  | 3 | 3 | **Case 28** | 62 | 3 | 33 | 0 |  | 0 | 0 |  |
|  | **Case 3** | 59 | 2 | 8 | 0 |  | 1 | 1 | **Case 29** | 55 | 3 | 11 | 1 |  | 0 | 1 |  |
|  | **Case 4** | 54 | 3 | 17 | 3 |  | 7 | 10 | **Case 30** | 43 | 2 | 35 | 1 |  | 0 | 1 |  |
|  | **Case 5** | 65 | 2 | 6 | 0 |  | 0 | 0 | **Case 31** | 44 | 2 | 18 | 0 | 1 | 0 | 1 |  |
|  | **Case 6** | 57 | 2 | 3 | 0 | 1 | 1 | 2 | **Case 32** | 53 | 3 | 38 | 0 |  | 0 | 0 |  |
|  | **Case 7** | 46 | 3 | 9 | 0 | 1 | 1 | 2 | **Case 33** | 60 | 3 | 17 | 1 |  | 1 | 2 |  |
|  | **Case 8** | 28 | 3 | 24 | 2 | 1 | 3 | 6 | **Case 34** | 30 | 1 | 34 | 0 |  | 0 | 0 |  |
|  | **Case 9** | 54 | 2 | 20 | 4 | 1 | 2 | 7 | **Case 35** | 42 | 3 | 34 | 0 |  | 0 | 0 |  |
|  | **Case 10** | 60 | 3 | 8 | 0 |  | 0 | 0 | **Case 36** | 34 | 3 | 11 | 1 | 1 | 1 | 3 |  |
|  | **Case 11** | 53 | 2 | 6 | 0 | 1 | 6 | 7 | **Case 37** | 28 | 2 | 35 | 0 |  | 0 | 0 |  |
|  | **Case 12** | 68 | 3 | 7 | 0 | 1 | 1 | 2 | **Case 38** | 63 | nr | 32 | 0 |  | 0 | 0 |  |
|  | **Case 13** | 44 | 3 | 6 | 0 |  | 0 | 0 | **Case 39** | 50 | 3 | 18 | 0 |  | 0 | 0 |  |
|  | **Case 14** | 61 | 2 | 24 | 0 |  | 8 | 8 | **Case 40** | 54 | 2 | 33 | 0 | 1 | 1 | 2 |  |
|  | **Case 15** | 77 | 2 | 2 | 1 |  | 1 | 2 | **Case 41** | 43 | 2 | 33 | 0 |  | 0 | 0 |  |
|  | **Case 16** | 49 | 3 | 4 | 0 | 1 | 0 | 1 | **Case 42** | 40 | 2 | 34 | 0 |  | 0 | 0 |  |
|  | **Case 17** | 52 | 3 | 5 | 0 |  | 1 | 1 | **Case 43** | 46 | 3 | 1 | 0 |  | 0 | 0 |  |
|  | **Case 18** | 49 | 3 | 6 | 0 |  | 1 | 1 | **Case 44** | 45 | 2 | 2 | 0 |  | 0 | 0 |  |
|  | **Case 19** | 42 | 2 | 27 | 0 |  | 1 | 1 | **Case 45** | 47 | 3 | 2 | 0 |  | 0 | 0 |  |
|  | **Case 20** | 67 | 2 | 21 | 0 |  | 0 | 0 | **Case 46** | 59 | nr | 5 | 0 |  | 0 | 0 |  |
|  | **Case 21** | 46 | 2 | 23 | 0 |  | 0 | 0 | **Case 47** | 45 | nr | 5 | 0 |  | 0 | 0 |  |
|  | **Case 22** | 57 | 3 | 29 | 0 | 1 | 0 | 1 |  |  |  |  |  |  |  |  |  |
|  | **Case 23** | 58 | 3 | 7 | 1 | 1 | 4 | 6 |  |  |  |  |  |  |  |  |  |
|  | **Case 24** | 46 | 3 | 23 | 0 | 1 | 0 | 1 |  |  |  |  |  |  |  |  |  |
|  | **Case 25** |  | nr |  | 0 | 0 | 1 | 1 |  |  |  |  |  |  |  |  |  |
|  | **Case 26** | 35 | 3 | 28 | 0 | 1 | 4 | 5 |  |  |  |  |  |  |  |  |  |

***ST: Storage time (Months), nr: not reported, MSI:** Microsatellite Instability **pLOH:** partial loss of heterozygosity, **cLOH: Complete** Loss of heterozygosity
